# Immobilized *Cerrena* sp. laccase: preparation, thermal inactivation, and operational stability in malachite green decolorization

**DOI:** 10.1038/s41598-017-16771-x

**Published:** 2017-11-27

**Authors:** Jie Yang, Zhengjuan Wang, Yonghui Lin, Tzi Bun Ng, Xiuyun Ye, Juan Lin

**Affiliations:** 1 Fujian Key Laboratory of Marine Enzyme Engineering, Fuzhou University, Fujian, 350116 China; 2 Technical Center, Fujian Entry-Exit Inspection and Quarantine Bureau, Fuzhou, Fujian, 350001 China; 3 School of Biomedical Sciences, Faculty of Medicine, The Chinese University of Hong Kong, Shatin, New Territories, Hong Kong, China

## Abstract

Laccases are polyphenol oxidases with widespread applications in various industries. In the present study, the laccase from *Cerrena* sp. HYB07 was immobilized with four methods, namely entrapment in alginate, covalently binding to chitosan as well as formation of cross-linked enzyme aggregates (CLEAs) and magnetic CLEAs (M-CLEAs). The activity recovery rates of the immobilized laccases ranged from 29% to 68%. Immobilization elevated the reaction temperature optimum and reduced substrate specificity, but not necessarily the turnover rate. pH stability of immobilized laccases was improved compared with that of the free laccase, especially at acidic pH values. Thermal inactivation of all laccases followed a simple first-order exponential decay model, and immobilized laccases displayed higher thermostability, as manifested by lower thermal inactivation rate constants and longer enzyme half-life time. Operational stability of the immobilized laccase was demonstrated by decolorization of the triphenylmethane dye malachite green (MG) at 60 °C. MG decolorization with free laccase was accompanied by a shift of the absorption peak and accumulation of a stable, colored intermediate tetradesmethyl MG, probably due to lower thermostability of the free laccase and premature termination of the degradation pathway. In contrast, complete decolorization of MG was achieved with laccase CLEAs at 60 °C.

## Introduction

Laccases comprise a class of multi-copper containing oxidases that are environmentally friendly and industrially important. Laccases have low substrate specificity and can break down or polymerize a wide range of compounds including recalcitrant dyestuffs and other organic contaminants, accompanied by concomitant reduction of molecular oxygen to water. Laccases have found applications in food, bioremediation, textile, biofuel and other industries^1–4^. However, laccase applications are hindered by high production costs, difficult recovery, and easy inactivation of the enzymes under industrial conditions. Lack of operational stability due to activity loss is another limiting factor for large-scale utilization of laccases^4–7^.

Laccases are immobilized to improve their stability, resistance, and reusability and to reduce application costs. Immobilization techniques include entrapment/encapsulation, adsorption, covalent binding and self-immobilization^5,8^. Entrapment is the physical retention of the enzyme in a porous solid matrix, such as frequently used calcium alginate. Entrapment is characterized by mass transfer limitations and low enzyme loading, although it does not rely on chemical interactions between the enzyme and support. Covalent binding is the most widely used method to link enzymes (often via lysine residues) with solid supports, but solid supports may also reduce the specific and volumetric activity of the biocatalyst. When chitosan is used as the support, enzymes can be either attached with a bifunctional cross-linker (e.g. glutaraldehyde) or conjugated directly via amide bond formation between activated carboxylic groups of the protein and nucleophilic groups of chitosan^9,10^. Formation of cross-linked enzyme aggregates (CLEAs) is an example of enzyme self-immobilization, or carrier-free immobilization. The procedure combines precipitation and immobilization in one step. When CLEAs are attached to magnetic nanoparticles, magnetic CLEAs (M-CLEAs) have higher mechanical strength than CLEAs and allow easy recycling of the enzyme with a magnetic field^11^. The variety of immobilization methods differ in the cost and ease of preparation, catalytic efficiency, stability and physical properties of the resultant enzymes, as well as the suitable application processes^7,12^. However, little comparison in these aspects has been made. Understanding the immobilization methods would benefit an informed choice of method.

Malachite green (MG) is a triphenylmethane dye commonly used in aquaculture to control protozoan and fungal infections of farmed fish. Because of the toxicity and mutagenicity of MG and its reduced form leucomalachite green, usage of MG is banned in many countries. Nonetheless, MG is still used in many places due to its efficacy, low cost and availability. MG is environmentally persistent, and laccase is a promising candidate in degradation and detoxification of MG^13^. *Cerrena* sp. HYB07 is a white-rot fungal strain that produces laccase with high yield, short production cycle, high specific activity and strong decolorizing ability^14^. Application of a purified HYB07 laccase, namely Lac7, in decolorization and detoxification of malachite green has been studied^13^. Lac7 is a major laccase produced by HYB07, as confirmed by MALDI-TOF MS/MS^14^; qRT-PCR analysis indicated that *Lac7* transcripts accounted for more than 99% of all transcripts of eight HYB07 laccase isozymes^15^. Purified Lac7 (in its free form) decolorizes approximately 90% of malachite green (100 mg/L) in 3 h at an ambient temperature of 28 °C, accompanied by reduced phytotoxicity. Furthermore, Lac7 converts MG via two simultaneous pathways without MG reduction to form leucomalachite green: one starts with successive *N*-demethylation, and the other one involves formation of MG carbinol and subsequent bond breakage between the central carbon and the *N*,*N*-dimethylamino phenyl ring^13^.

In the present study, four methods, namely entrapment, covalent binding, CLEAs and M-CLEAs, were employed to immobilize *Cerrena* sp. HYB07 laccase. The crude laccase, consisting predominantly of Lac7, was used for immobilization and characterization because the crude enzyme is more economical and practical in real applications than the purified enzyme. The immobilized laccases were compared in terms of activity recovery rates, kinetics, thermal inactivation parameters, etc. The advantage of the immobilized laccase was manifested by stable performance in malachite green decolorization without unwanted accumulation of a colored intermediate.

## Materials and Methods

### Laccase

*Cerrena* sp. HYB07 laccase was used in this study. Fermentation of *Cerrena* sp. HYB07 was carried out as previously described^16^, and the fermentation broth was harvested by paper filtration and centrifugation at 8,000 g for 5 min. The resulting crude enzyme preparation was referred to as the free laccase.

### Materials

2,2′-azino-bis (3-ethylbenzothiazoline-6-sulfonic acid) (ABTS) and MG were purchased from Sigma-Aldrich, USA. Glutaraldehyde, chitosan and salts were purchased from Sinopharm Chemical Reagent Co., Ltd., China. All chemicals were of analytical grade (or the highest purity available).

### Assay methods

Protein content was quantified with BCA Protein Assay Kit (Pierce, USA). Laccase activity was assayed with ABTS. Absorbance change at 420 nm was followed for 5 min. One unit of enzyme activity was defined as the amount of enzyme needed to oxidize 1 μmol ABTS in 1 min. All measurements were carried out in triplicate.

### Laccase immobilization

Four methods were used to immobilize laccase. The immobilization parameters were optimized to maximize enzyme activity recovery. CLEAs and M-CLEAs were prepared according to the reported protocols^16,17^. Briefly, the pH value of the fermentation broth was adjusted to 8.0 before immobilization. For CLEAs, laccase was precipitated with (NH_4_)_2_SO_4_ at 25 °C for 2 h, and crosslinking was carried out with 30 mM glutaraldehyde for 3 h. For M-CLEAs, amino-functionalized magnetic nanoparticles (MNPs) were added at the laccase:MNPs ratio of 0.8:1, and 40 mM glutaraldehyde was used to crosslink laccase for 2 h.

For laccase entrapment, alginate was dissolved in distilled water at a concentration of 2% (w/v) at 60 °C. Enzyme broth was mixed with alginate, and the mixture was added dropwise to 2% (w/v) CaCl_2_ by using a 0.4-mm-diameter syringe to produce beads. The alginate beads were harvested by filtration, and washed several times with distilled water until no laccase activity was detected in washed water.

Covalent binding of laccase to chitosan was carried out as previously described^18^ with minor modifications. Chitosan was dissolved in 1% (v/v) acetic acid at the ratio of 3% (w/v) and added dropwise to 2 M NaOH to form spherical beads. The beads were subsequently harvested by filtration, and washed several times with distilled water until a neutral pH value was reached. Chitosan beads (1 g) were added to 5 mL 0.4% glutaraldehyde, crosslinked for 16 h and washed with distilled water. Laccase was immobilized onto glutaraldehyde-crosslinked chitosan beads at the ratio of 1:1 (v/w) at room temperature for 8 h.

### Kinetic parameters of laccases

Kinetic studies were carried out in triplicate with 2.5–500 μM ABTS at the respective optimum pH and temperature of each laccase. The kinetic parameters were estimated by nonlinear regression of the Michaelis-Menten equation with GraphPad Prism version 5.0 (GraphPad Software, Inc., USA).

### Effect of pH on activity and stability of laccases

The effect of pH on laccase activity was determined from pH 2.0 to 7.0 at 30 °C. The maximum enzyme activity was set as 100% for calculation of relative enzyme activity. pH stability was studied by incubating the enzyme at pH 2.0–10.0 at 30 °C for 12 h, and the residual enzyme activity was quantified at the optimal pH and temperature. Activity of the untreated enzyme was set as 100%. Glycine-HCl buffers (pH 2.0–3.0), citrate-phosphate buffers (pH 3.0–6.5), sodium phosphate buffers (pH 7.0–8.0) and glycine-NaOH buffers (pH 9.0–10.0) were used. All measurements were performed in triplicate.

### Effect of temperature on activity and stability of laccases

To ascertain the optimum temperature, laccase activity was measured at the optimum pH and temperatures from 20 to 70 °C. Thermostability was analyzed by incubating the enzyme at three different temperatures over a period of 6 h. A sample was taken regularly to assay the residual activity. All measurements were performed in triplicate.

The experimental data were fitted to a simple first-order exponential decay model^19,20^:

1 $${\rm{E}}\,\mathop{\longrightarrow }\limits^{k}{{\rm{E}}}_{{\rm{i}}{\rm{n}}}$$

where E and E_in_ represent the enzyme before and after heat inactivation, respectively. The thermal inactivation parameters *k* and *t*_1/2_ were calculated by nonlinear regression with GraphPad Prism version 5.0 (GraphPad Software, Inc., USA). Enzyme residual activity could be expressed as a function of time by the following equation:

2 $$A={{\rm{e}}}^{-kt}$$

where *A* is the residual enzyme activity, *k* is the inactivation rate constant, and *t* is the time. The half-life time (*t*_1/2_) was determined by using the following equation^19^:

3 $${t}_{1/2}=\frac{0.693}{k}$$

The free energy for thermal inactivation (Δ*G*°) was calculated from the inactivation rate constant *k* as follows:

4 $${\rm{\Delta }}{G}^{O}=-RT\,\mathrm{ln}(\tfrac{kh}{KT})$$

where *R* is the universal gas constant (8.314 J mol^−1^ K^−1^), *T* is the temperature (K), *h* is the Planck constant (6.626 × 10^−34^ J s) and *K* is the Boltzmann constant (1.38 × 10^−23^ J K^−1^).

### Decolorization of MG with laccases

Decolorization of MG was carried out at 30 and 60 °C. The starting reaction mixture contained 50 mM citrate-phosphate buffer (pH 6.0), 100 mg/L MG and 3 U/mL laccase. The mixture with heat-inactivated laccase was used as the negative control. After decolorization, the reaction mixtures were subjected to UV-visible analysis with a UV-Vis spectrophotometer (U-2910, Hitachi, Japan). Decolorization efficiency was monitored at 618 nm and calculated with the following formula:

5 $${\rm{Decolorization}}\,{\rm{efficiency}}\,( \% )=\frac{{\rm{A0}}-{\rm{A1}}}{{\rm{A0}}}\times 100$$

where A_0_ and A_1_ are the absorption of MG before and after laccase treatment, respectively.

Degradation products were identified with liquid chromatography-time of flight mass spectrometry (LC-TOF MS) as previously described^13^. Briefly, an HPLC system (Agilent 1200 Series, equipped with a Phenomenex Luna C-18 analytical column) coupled with Agilent 6224 Accurate-Mass TOF MS was used. The column effluent was introduced into the electrospray ionization source of the mass spectrometer in positive ion mode. LC-TOF MS accurate mass spectra were recorded across the range 70–400 *m*/*z*. Data processing was carried out by using Applied Biosystems/MDS-SCIEX Analyst QS software (Frankfurt, Germany) with accurate mass application-specific additions from Agilent MSD TOF software.

## Results and Discussion

### Laccase immobilization by four methods

Laccase was immobilized via entrapment in alginate beads, covalent binding to chitosan beads and aggregation as CLEAs and M-CLEAs under optimized conditions. Differences in activity recovery were observed, which should be accounted for by the immobilization methods since the same laccase was used. Activity recovery is an important factor governing the cost of the immobilized enzyme. Laccase CLEAs had the highest recovery rate (~68%) of all immobilized enzymes, followed by chitosan-bound laccase (~50%) and laccase M-CLEAs (~47%), and entrapment resulted in the lowest recovery rate of approximately 30% (Table 1). Our activity recovery rate of the entrapment method was lower than the immobilization efficiency of *Coriolopsis gallica* ^21^, whereas the activity recovery of chitosan-bound laccase was similar to the one reported for *T. versicolor* laccase^22^. Although the preparation of CLEAs and M-CLEAs has been reported in our previous work^16,17^, the effects of pH and temperatures on the immobilized laccases were characterized in the present study.

**Table 1:** Comparison of free and immobilized laccases.

Laccase	Activity recovery (%)	Optimal pH	Optimal temperature (°C)	*K*_m_ (μM)	*V*_max_ (mM/min/mg)
Free	—	3.0	40	25.9	123.0
CLEAs	68.1	3.0	50	37.5	171.4
M-CLEAs	46.8	3.0	50	52.4	111.2
Entrapped	29.8	3.0	50	42.5	16.9
Chitosan-bound	50.1	3.0	50	52.5	8.3

### Effect of pH on activity and stability of laccases

The optimal pH of the free laccase was 3.0, and so was the optimal pH of the immobilized laccases (Table 1). The immobilized laccases had wider pH activity ranges compared with the free laccase. At pH 2.0, the free laccase was nearly completely inactive, but the immobilized laccases had 20-60% remaining activity, with the entrapped laccase being the most active. At pH 5.0, all immobilized laccases had over 50% relative activity, whereas the free laccase had only 12% relative activity.

**Figure 1:**
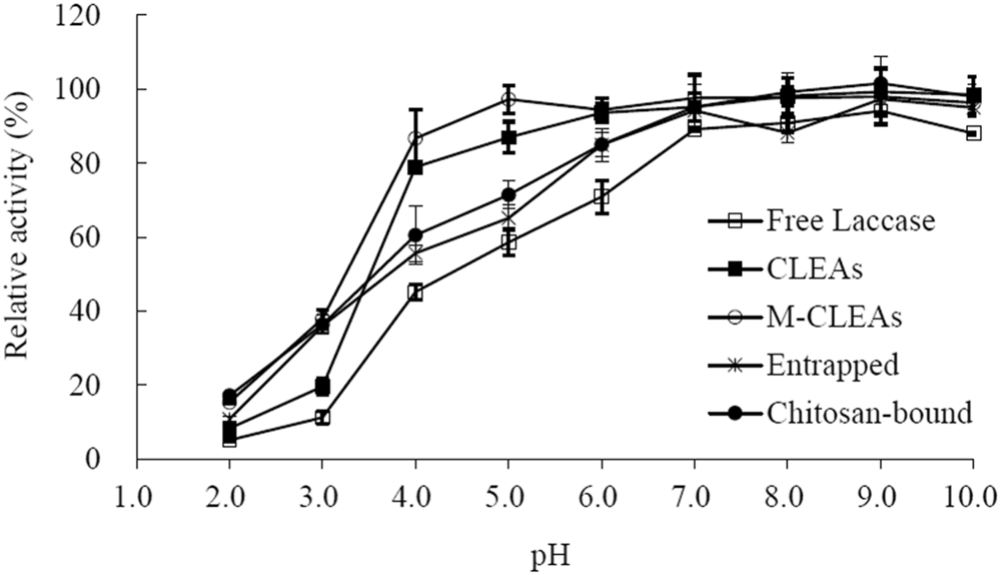
Effect of pH on stability of free and immobilized laccases.

The free laccase was stable at pH 7.0 and above; after incubation at 30 °C for 12 h, over 80% of the original enzyme activity was observed. Immobilization improved laccase stability over the tested pH range, and M-CLEAs were the most stable. At pH 4.0, M-CLEAs retained 86% enzyme activity, which was 2-fold of the remaining activity of the free laccase; in comparison, CLEAs retained 79% residual activity, followed by chitosan-bound laccase and entrapped laccase. All laccases were unstable at acidic pH values 2.0 and 3.0 (Fig. 1).

### Effect of temperature on activity of laccases

The free laccase and the immobilized laccases were most active at 40 and 50 °C, respectively (Table 1); immobilization increased the temperature optimum by 10 °C. At 70 °C, the free laccase had only a relative activity of 15%, but the immobilized laccases had a relative activity above 30%, with M-CLEAs displaying the highest relative activity of 56%. At 20–30 °C, the entrapped laccase had the lowest relative activity.

### Kinetic parameters of laccases

Immobilization led to lower substrate (ABTS) affinity, as seen in higher *K*_m_ values (Table 1). CLEAs had the lowest *K*_m_ value of all immobilized laccases, whereas M-CLEAs and chitosan-bound laccases had the lowest substrate affinity with the *K*_m_ values being two-fold of that of the free enzyme. The *V*_max_ value of CLEAs (171.4 mM/min/mg) was higher than that of the free laccase (123.0 mM/min/mg), followed by M-CLEAs with a *V*_max_ value (111.2 mM/min/mg) slightly lower than that of the free enzyme. Immobilization sometimes improves the catalytic activity of laccases^23–26^ despite the common concern of reduced enzyme flexibility, steric hindrance and diffusion limitations^11,27^. The other two carrier-based immobilized laccases had *V*_max_ values one magnitude lower than that of the free enzyme. This was not surprising since carrier-binding and entrapment methods have the disadvantages of generating large non-catalytic mass and diminishing the efficiency of the enzyme^6^.

### Thermostability of laccases

**Figure 2:**
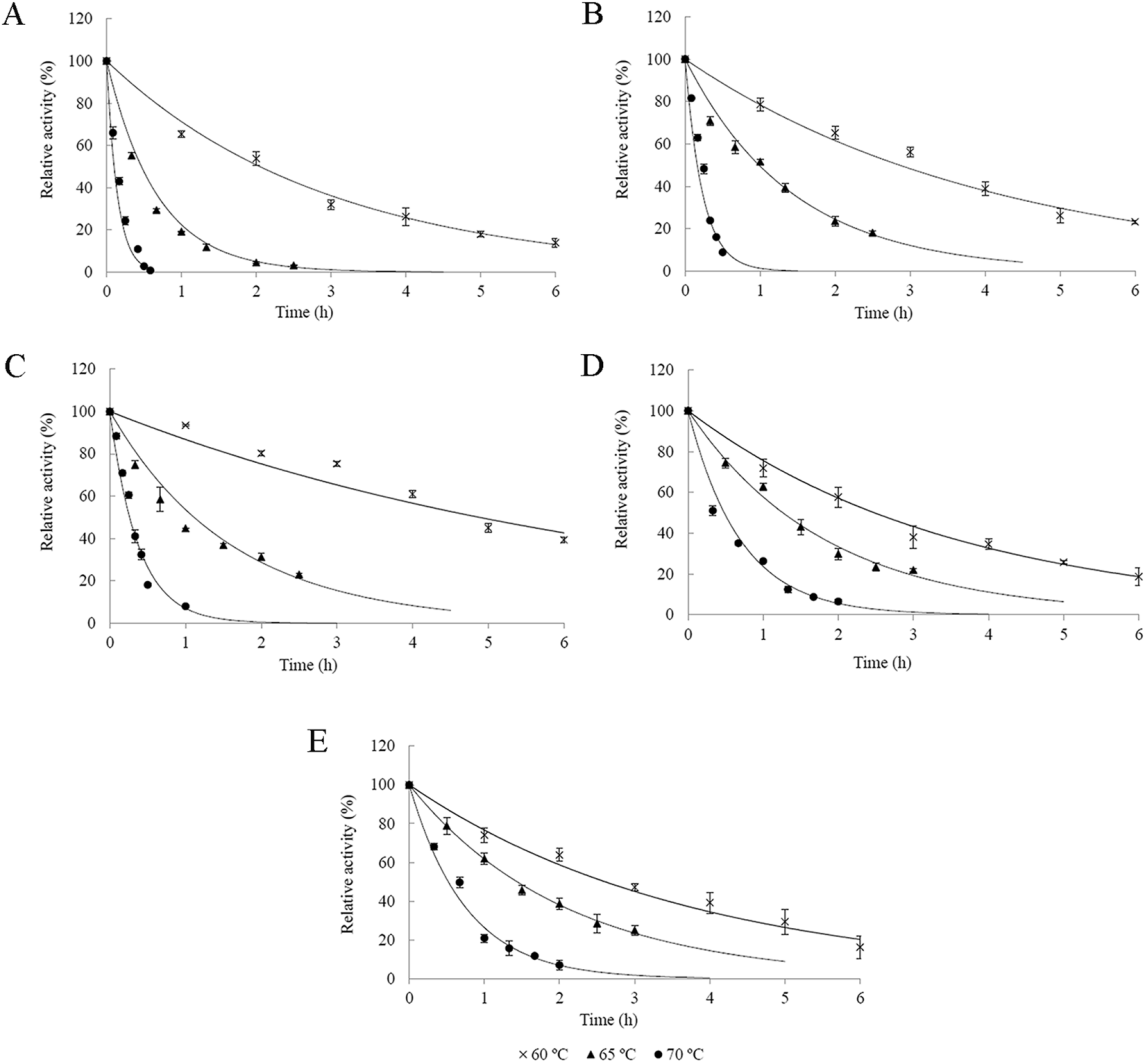
Thermostability of free and immobilized laccases. (**A**) Free laccase; (**B**) laccase CLEAs; (**C**) laccase M-CLEAs; (**D**) entrapped laccase; (**E**) chitosan-bound laccase.

Thermostability of free and immobilized laccases was studied at 60–70 °C. Thermal inactivation of all laccases followed a simple first-order exponential decay model (Fig. 2), implying a single step transition from the active (E) to denatured (E_in_) state, either through breakage of a key bond or deformation of an important structure^20^. The thermal inactivation parameters are presented in Table 2. Regardless of the form of laccase, with increasing temperatures, inactivation rate constant *k* increased significantly, accompanied by shorter *t*_1/2_. Immobilization improved thermostability of laccase, as manifested by lower *k* values and longer *t*_1/2_ of immobilized laccases. Interestingly, although laccase M-CLEAs were more stable at 60 °C than entrapped and covalently bound laccases, the latter two were more tolerant of higher temperatures (Table 2).

**Table 2:** Thermal inactivation parameters of free and immobilized laccases.

Laccase	T (°C)	*k* (h^−1^)	*t*_1/2_ (h)	*R* ^2^	Δ*G*° (kJ/mol)
Free	60	0.35	1.99	0.990	100.34
	65	1.73	0.40	0.998	97.40
	70	5.42	0.13	0.995	95.63
CLEAs	60	0.23	2.99	0.984	101.51
	65	0.74	0.93	0.960	99.79
	70	3.63	0.19	0.949	96.77
M-CLEAs	60	0.15	4.73	0.926	102.69
	65	0.67	1.04	0.948	100.07
	70	2.53	0.27	0.954	97.80
Entrapped	60	0.29	2.42	0.991	100.87
	65	0.55	1.26	0.990	100.62
	70	1.58	0.44	0.983	99.14
Chitosan-bound	60	0.25	2.75	0.986	101.28
	65	0.49	1.42	0.997	100.95
	70	1.28	0.54	0.984	99.74

The free energy of the inactivation process Δ*G*° values are all positive, indicating thermal inactivation was nonspontaneous. The decrease in Δ*G*° values with increasing temperatures implies a progressive destabilization of the enzyme molecule. Similarly, immobilized laccases had higher Δ*G*° values than the free laccase. Variation in Δ*G*° for all laccases was less than 5 kJ/mol; little variance was seen with entrapped and chitosan-bound laccases (Table 2), consistent with high stability of these two immobilized laccases. Despite many reported improvements of thermostability upon immobilization, not all immobilized laccases were more resistant to thermal inactivation than their free counterparts. For example, laccase immobilized on modified silica showed similar thermostability to the free enzyme^19^, and laccase immobilized on multi-walled carbon nanotubes was even less thermostable with greater *k*, shorter *t*_1/2_ and smaller Δ*G* values^28^.

### Decolorization of MG by laccases

We have previously shown efficient MG decolorization by purified Lac7 (the predominant HYB07 laccase isozyme) at 28 °C; with complete decolorization, the absorbance peak of MG at 618 nm disappeared without appearance of new peaks. In the meantime, transformation products identified by LC-TOF MS suggested two alternative degradative pathways of MG by laccase. In the first pathway, MG (*m*/*z* 329.20) undergoes four consecutive demethylation steps to form successively, desmethyl MG (*m*/*z* 315.19), didesmethyl MG (*m*/*z* 301.17), tridesmethyl MG (*m*/*z* 287.16), and finally tetradesmethyl MG (*m*/*z* 273.14), which then undergoes further degradation. In the second pathway, MG is first hydroxylated to form MG carbinol, which is subsequently broken down to *N*,*N*-dimethylaniline and (dimethyl amino-phenyl)-phenyl methanone (*m*/*z* 226.12). The latter is then demethylated to (methyl amino-phenyl)-phenyl methanone (*m*/*z* 212.11) and (amino phenyl)-phenyl methanone (*m*/*z* 198.09), which then undergoes further degradation^13^.

**Figure 3:**
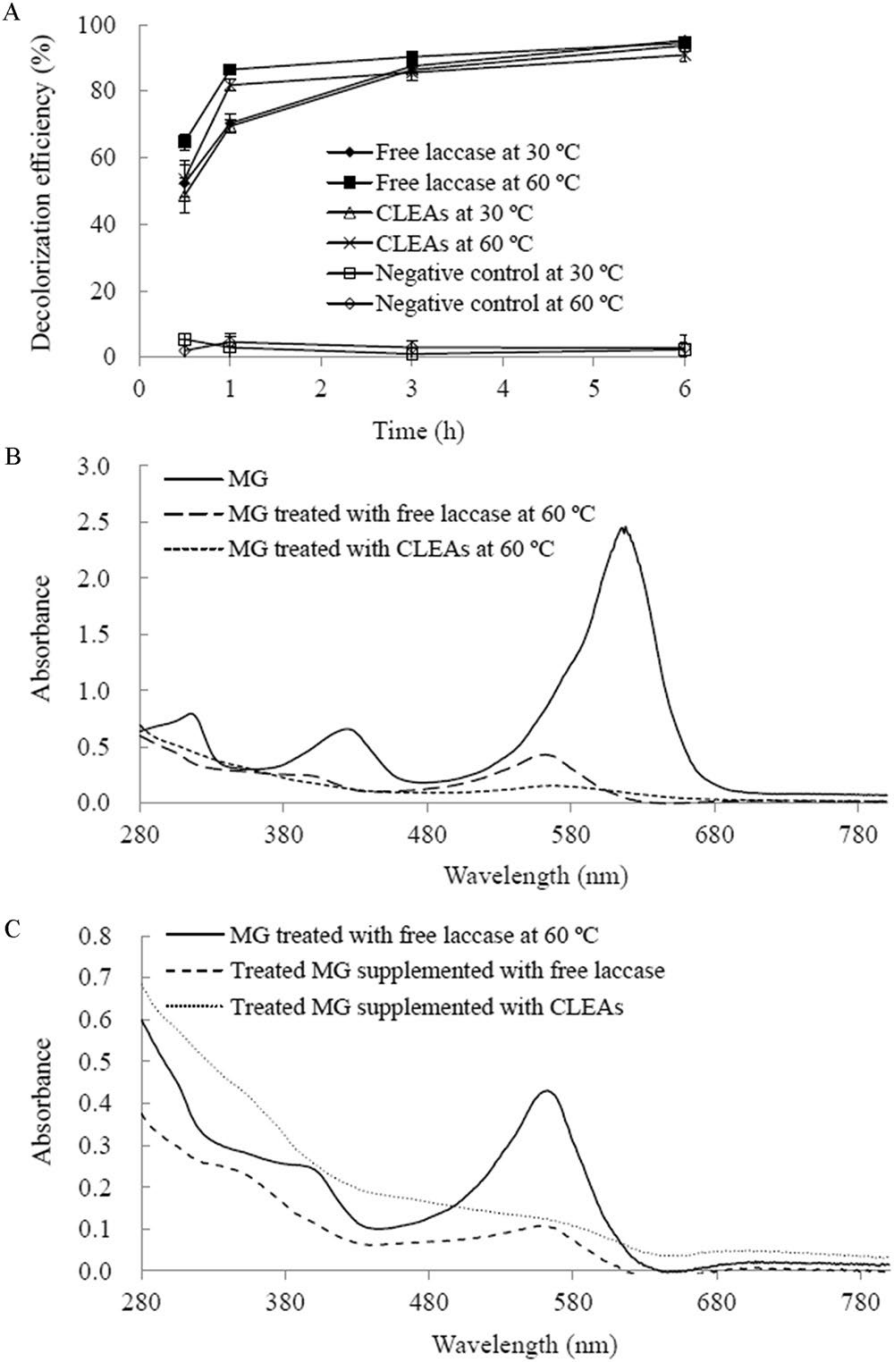
Decolorization of MG by free and immobilized laccases. (**A**) Efficiencies of MG decolorization by free laccase and laccase CLEAs. (**B**) UV-vis spectra of MG before and after decolorization by free laccase at 30 and 60 °C. (**C**) UV-vis spectra of treated MG supplemented with free and immobilized laccases. MG treatment was carried out at 60 °C.

**Figure 4:**
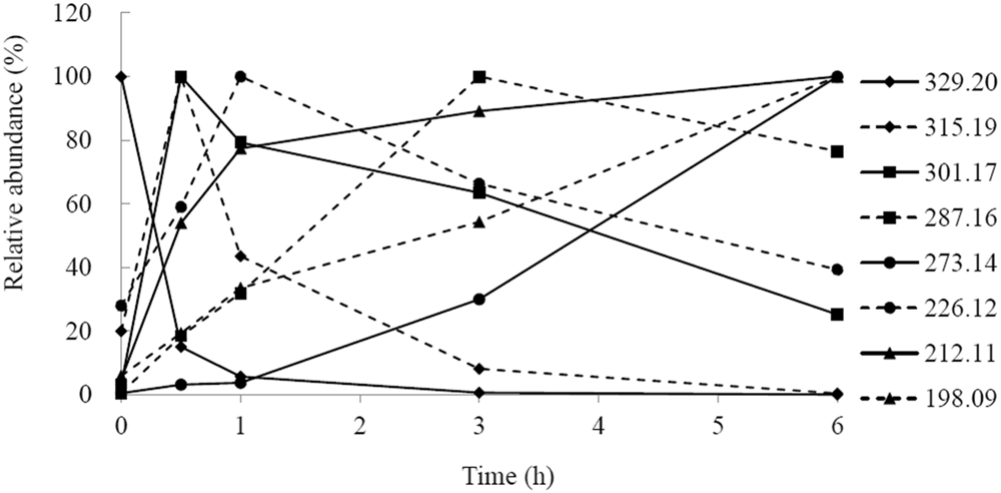
Time course of MG and the transformation products during treatment with free laccase at 30 °C. MG (*m*/*z* 329.20) and the transformation products (*m*/*z* 315.19, 301.17, 287.16, 273.14, 226.12, 212.11 and 198.09) were detected with LC-TOF MS. The highest level of MG or each transformation product during the treatment was set to 100%.

In this work, crude HYB07 laccase was used and displayed MG decolorization efficiency close to that of purified Lac7 (approximately 90% MG decolorization after 3 h at the ambient temperature), which was expected considering Lac7 was the most abundant laccase isozyme^14,15^. Furthermore, similar MG decolorization efficiencies were obtained for the free laccase and laccase CLEAs at 30 and 60 °C (Fig. 3A). The negative controls did not show significant MG decolorization. At 60 °C, MG decolorization was accelerated compared to that at 30 °C, but surprisingly, MG treatment with the free laccase at 60 °C led to the formation of a persistent light pink color, corresponding to a new absorbance peak at 560 nm (Fig. 3B). This new color was not seen with the free laccase treatment at 30 °C nor the treatments with laccase CLEAs at both temperatures. Replacing the crude HYB07 enzyme with free, purified Lac7 also resulted in the residual color and absorbance peak shift at 60 °C. We were prompted to decipher the reason behind the residual color of the free laccase treatment at 60 °C. All seven MG transformation products as we previously reported for Lac7-mediated MG degradation^13^, were identified by LC-TOF MS in all laccase treatments (Fig. 4), suggesting that the free and immobilized HYB07 laccases catalyzed the same MG transformation pathways, regardless of the reaction temperature.

**Figure 5:**
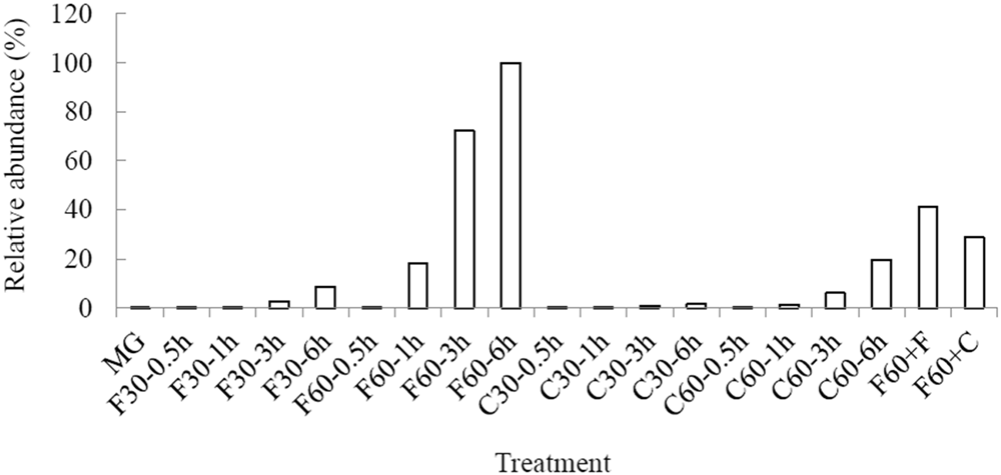
Accumulation of tetradesmethyl MG (*m*/*z* 273.14) during MG degradation by free and immobilized laccases at 30 and 60 °C. Tetradesmethyl MG (*m*/*z* 273.14) was detected with LC-TOF MS. The highest level of tetradesmethyl MG (*m*/*z* 273.14) was observed in MG treated with free laccase at 60 °C for 6 h and was set to 100%. For the laccase treatments of MG, the first letters “F” and “C” indicate free laccase and CLEAs, respectively. The number after the first letter indicates the treatment temperature, and the number after “−” indicates treatment time. After treating MG with free laccase at 60 °C for 6 h, the solution was supplemented with free laccase (F60+F) or CLEAs (F60+C) and incubated overnight.

Since the new color and the absorbance peak at 560 nm in the decolorization mixture with free laccase at 60 °C were characteristic of tetradesmethyl MG (m/z 273.14)^29^, a stable intermediate of MG demethylation during laccase transformation (Fig. 4)^13^, we speculated that since the free laccase was inactivated more quickly at 60 °C than at 30 °C, the lower stability of the free laccase at 60 °C led to unexpected interruption of the degradation pathway(s), accumulation of the colored metabolite and thus incomplete decolorization. On the other hand, at the lower temperature of 30 °C, laccase was able to slowly, but steadily reach complete decolorization of MG. Similarly, with enhanced thermostability and operational stability, laccase CLEAs could decolorize MG without abrupt termination of the degradation process or forming a new color. Supporting our hypothesis, LC-TOF MS analysis demonstrated abundance of tetradesmethyl MG during MG decolorization by free laccase at 60 °C, but not in the other treatments. Consistently, when the colored reaction mixture was supplemented with fresh laccase, either free laccase or CLEAs, and incubated overnight at 60 °C, the amount of tetradesmethyl MG was reduced (Figs 3C and 5).

Immobilized laccases used in dye decolorization often display superior performance to the free laccase with respect to decolorization efficiency, reusability and resistance to inhibitors. Examples of such immobilized laccases include *Coriolopsis gallica* laccase entrapped in alginate beads^21^, *Trametes pubescens* ^18^ and *T. versicolor* ^30^ laccases bound to chitosan beads, *Cerrena* sp. HYB07^16^ and *Shewanella putrefaciens* ^26^ laccase CLEAs and *T. versicolor* laccase M-CLEAs^24^. In the present study, we dissected interrupted malachite green degradation caused by quick inactivation of the free laccase at 60 °C and showed that the immobilized laccase offered operational stability at higher temperatures, which was presumably due to its higher thermostability. Unlike the free laccase, the immobilized laccase achieved complete decolorization of MG as expected, without accumulation of tetradesmethyl MG at 60 °C. The significance of improved laccase stability certainly goes beyond laccase-catalyzed malachite green decolorization and is appreciated in many processes that may require higher temperatures, such as denim bleaching^31^, delignification^32^, pulp bleaching^33^, organic synthesis^34,35^, bioremediation^36^, etc. Nonetheless, the comparison of malachite green decolorization behavior of the free and immobilized laccases intuitively illustrated the significance of enhanced operational stability of the immobilized enzyme.

## Conclusions

Four types of immobilized laccases, namely entrapped, chitosan-binding, CLEAs and M-CLEAs, were prepared and compared in terms of activity recovery, pH and temperature optima, kinetics and thermostability. In particular, laccase CLEAs offered predictable decolorizing performance compared with the free laccase. At 60 °C, treatment of MG with the free laccase, but not laccase CLEAs, resulted in premature termination of degradation and accumulation of a stable, colored intermediate, tetradesmethyl MG. This study serves as an example that immobilization improves thermostability and operational stability of laccases and facilitates their applications.
